# Encoding of tactile stimulus parameters by mechanosensory P cells of the medicinal leech *Hirudo medicinalis*

**DOI:** 10.1186/1471-2202-12-S1-P180

**Published:** 2011-07-18

**Authors:** Friederice Pirschel, Jutta Kretzberg

**Affiliations:** 1 Computational Neuroscience, Institute of Biology and Environmental Sciences, University of Oldenburg, D-26111 Oldenburg, Germany

## 

Some nervous systems achieve precise behavioral responses to sensory stimuli with surprisingly few neurons. The medicinal leech possesses one of the smallest central nervous systems with ~10.000 neurons with ganglia containing up to 400 neurons. Individual ganglia are able to elicit behavioral responses to sensory stimuli, e.g. bending away from pressure applied to the skin, even if they are dissected from the rest of the CNS. The local bend reflex [1] is initiated by activation of the mechanosensory P (‘pressure’) cells, processed by one layer of interneurons and one layer of motorneurons, which cause muscle contraction and elongation. This behavioral response of the leech was shown to depend on stimulus location and intensity [2]. Based on responses of only two P cells with overlapping receptive fields, the leech can discriminate locations of tactile stimuli which are only 9° of the body circumference apart [3].

In order to investigate how P cells of the medicinal leech encode information about tactile stimuli, we have recorded intracellularly from P cells while stimulating the skin mechanically with tactile stimuli of varied intensity and duration. The neuronal responses were analyzed with respect to their features latency, spike count and interspike intervals by performing stimulus estimation based on the maximum likelihood method.

We found that the intensity of tactile stimuli influenced all response features considered in this study. With rising intensity the spike count increased, whereas the latency and the interspike intervals (in particular the 1^st^ ISI) decreased. Longer stimulus duration led to a greater spike count, while the other response features were basically unaffected. When estimating seven different pressure intensities between 5mN and 60mN based on the spike count, the latency, or the 1^st^ ISI of individual P cell responses, all three response features led on average to similar estimation performances (~34% correct estimations, chance level 14.3%, 10 P cells). The 2nd and 3rd interspike intervals yielded slightly lower performances (~28% correct estimations). Combinations of two response features did not improve the estimation performances.

Since stimulus duration strongly affected spike count, combinations of three stimulus durations and two pressure intensities could be estimated much better based on spike counts (~61% correct estimations, chance level 16.7%, 12 P cells) than on the other response features or their combinations (between ~22% and ~35% correct estimations).

These findings suggest that stimulus duration is encoded by spike count, while spike count, interspike intervals and latency of single P cells provide at least to some extend redundant information about stimulus intensity. However, a related study (Kappel, Pirschel, Kretzberg, this issue) revealed that interneuron EPSPs are more strongly influenced by the 1^st^ ISI than by spike count of P cell responses. Most probably, the leech CNS combines the responses of the two P cells with overlapping receptive fields and the responses of two T (‘touch’) cells to reliably estimate stimulus intensity, as it was found for stimulus location [3].
